# Wastewater-Based SARS-CoV-2 Surveillance in Northern New England

**DOI:** 10.1128/spectrum.02207-21

**Published:** 2022-04-12

**Authors:** Diana M. Toledo, Ashlee A. Robbins, Torrey L. Gallagher, Kenneth Chase Hershberger, Rachael E. Barney, Sabrina M. Salmela, Davey Pilcher, Mark A. Cervinski, Robert D. Nerenz, Zbigniew M. Szczepiorkowski, Gregory J. Tsongalis, Joel A. Lefferts, Isabella W. Martin, Jacqueline A. Hubbard

**Affiliations:** a Department of Pathology and Laboratory Medicine, Dartmouth-Hitchcock Medical Center, Geisel School of Medicine at Dartmouth, Hanover, New Hampshire, USA; b The Broad Institute at MIT and Harvard, Cambridge, Massachusetts, USA; University of Mississippi Medical Center

**Keywords:** COVID-19, PCR, RNA, RT-ddPCR, RT-qPCR, SARS-CoV-2, wastewater, wastewater treatment

## Abstract

SARS-CoV-2 viral RNA is shed in the stool of 55–70% of infected individuals and can be detected in community wastewater up to 7 days before people present with COVID-19 symptoms. The detection of SARS-CoV-2 RNA in wastewater may serve as a lead indicator of increased community transmission. Here, we monitored viral concentrations in samples collected from nine municipal wastewater facilities in New Hampshire (NH) and Vermont (VT).Twenty-four-h composite primary influent wastewater samples were collected from nine municipal wastewater treatment facilities twice per week for 5 months (late September 2020 to early February 2021). Wastewater was centrifuged for 30 min at 4600 × *g*, then the supernatant was frozen until further analysis. Once thawed, samples were concentrated, extracted, and tested for SARS-CoV-2 RNA using reverse transcriptase-quantitative PCR (RT-qPCR) and reverse transcriptase-droplet digital PCR (RT-ddPCR) detection methods. Active case counts for each municipality were tracked from the NH and VT state COVID-19 dashboards. We received a total of 283 wastewater samples from all sites during the study period. Viral RNA was detected in 175/283 (61.8%) samples using RT-qPCR and in 195/283 (68.9%) samples using RT-ddPCR. All nine sites showed positivity in the wastewater, with 8/9 (88.8%) sites having over 50% of their samples test positive over the course of the study. Larger municipalities, such as Nashua, Concord, and Lebanon, NH, showed that SARS-CoV-2 positivity in the wastewater can precede spikes in active COVID-19 case counts by as much as 7 days. Smaller municipalities, such as Woodsville, NH and Hartford, VT, showed sporadic SARS-COV-2 detection and did not always precede a rise in active case counts. We detected SARS-CoV-2 RNA in samples from all 9 municipalities tested, including cities and small towns within this region, and showed wastewater positivity as an early indicator of active case count increases in some regions. Some of the smaller rural municipalities with low case counts may require more frequent sampling to detect SARS-CoV-2 in wastewater before a case surge. With timely collection and analysis of wastewater samples, a community could potentially respond to results by increasing public health initiatives, such as tightening mask mandates and banning large indoor gatherings, to mitigate community transmission of SARS-CoV-2.

**IMPORTANCE** Despite vaccination efforts, the delta and omicron variants of SARS-CoV-2 have caused global surges of COVID-19. As the COVID-19 pandemic continues, it is important to find new ways of tracking early signs of SARS-CoV-2 outbreaks. The manuscript outlines how to collect wastewater from treatment facilities, concentrate the virus in a dilute wastewater sample, and detect it using two sensitive PCR-based methods. It also describes important trends in SARS-CoV-2 concentration in wastewater of a rural region of the United States from Fall 2020 – Winter 2021 and demonstrates the utility of wastewater monitoring as a leading indicator of active SARS-CoV-2 cases. Monitoring changes in concentration of SARS-CoV-2 virus in wastewater may offer an early indicator of increased case counts and enable appropriate public health actions to be taken.

## INTRODUCTION

The SARS-CoV-2 virus was discovered as the cause of the novel Coronavirus Disease-19 (COVID-19) in late 2019 and resulted in a global pandemic with over 200 million people infected with the virus and 4.3 million COVID-19-related deaths worldwide as of August 2021 (1). COVID-19 has mostly been monitored in the population by testing symptomatic individuals for the presence of SARS-CoV-2 RNA in an upper respiratory-derived sample (clinical testing) (2). Some surveillance testing of asymptomatic individuals with early contact tracing was conducted, but was limited by the rapid spread of the virus and testing capacity of most clinical and public health laboratories. Testing of symptomatic individuals is a lagging indicator of the virus’ community transmission and progression. This is due to symptoms taking 3 days to 2 weeks to present in a given individual (3, 4), depending on the viral variant, and the time interval between sample collection and result reporting. While testing accessibility has improved since the beginning of the pandemic, staffing shortages, supply chain issues, and the surge of the omicron variant in late 2021 coupled with winter holiday travel and gatherings caused turnaround times to rise again. In addition, many individuals do not seek PCR testing because of the increased use of at-home rapid antigen testing, vaccination uptake, and upsurge in cases with milder symptoms, making it difficult to estimate viral spread in the community. An alternative method for early detection and tracking COVID-19 community spread could help mitigate transmission.

Wastewater viral surveillance has been performed within the last century to monitor for poliovirus (5), hepatitis A virus (6), and norovirus outbreaks (6). As early as the 1940s, cell culture methods were used to monitor sewage for poliovirus, followed by the more sensitive method of PCR (PCR) in the 1990s to today. Many viruses, including coronaviruses, can be detected in both excrement and wastewater (7). During the early global rise of COVID-19 infections in early 2020, several studies found that SARS-CoV-2 viral RNA is present in approximately 55–70% of human stool samples from infected individuals (8, 9). SARS-CoV-2 in stool remains detectable for several weeks after infection, and is thought to peak at 14–18 days (10, 11). Fecal SARS-CoV-2 concentration varies significantly, but it has been reported to be as high as 10^8.1^ copies/g feces (10, 11). While the exact survival time of the SARS-CoV-2 virus in feces is unknown, other coronaviruses survived 1 h to 4 days, depending on the type and pH of the stool (12). The SARS-CoV-2 detection window in wastewater is temperature-sensitive and can range from 2 days at 20°C and up to 14 days at 4°C (13). Detecting SARS-CoV-2 in wastewater might help gauge the prevalence of SARS-CoV-2 in a region.

There were several key early studies focusing on the detection the SARS-CoV-2 virus in samples from wastewater treatment facilities (WWTF). A proof-of-concept study from Australia showed that SARS-CoV-2 RNA was detected using reverse transcriptase real-time PCR (RT-qPCR) in concentrated untreated wastewater from pump stations and treatment plants (14). Some studies in the United States were conducted in early to mid-2020, but they focused on larger metropolitan areas such as Boston, Massachusetts (15), and New Haven, Connecticut (16). SARS-CoV-2 was detected at concentrations of 57–303 copies/mL in wastewater from the Boston treatment plant in mid-March 2020 (15). In New Haven, SARS-CoV-2 concentration in sludge indicated a rise in active cases 1–4 days earlier than the associated rise in hospital admissions and 6–8 days sooner than the rise in reported positive clinical test results in the context of delayed testing (16). Therefore, SARS-CoV-2 RNA may be detected in community wastewater several days before current key indicators, such as hospital visits and diagnostic test results, and could function as an alarm system for near-future community spread.

The detection method used may affect the sensitivity, turnaround time, and subsequent utility of this type of environmental monitoring. Two different PCR-based methods have been routinely used for SARS-CoV-2 detection: reverse transcriptase-quantitative PCR (RT-qPCR) and reverse transcriptase-droplet digital PCR (RT-ddPCR). Both methods include a reverse transcription step to convert viral RNA into complementary DNA (cDNA) to be used for downstream PCR amplification. During qPCR, fluorescence in the entire well is measured concurrently with sample amplification, and one cycle threshold (Cq) is determined for each well. During ddPCR, the sample is initially fragmented into ∼10,000 – 20,000 droplets that subsequently undergo PCR amplification, followed by a fluorescence reading of each individual droplet allowing for absolute quantification. Due to thousands of PCRs occurring during ddPCR, it is considered more sensitive than qPCR in the detection of SARS-CoV-2, especially at lower viral loads (17). This unique longitudinal study used two different detection methods to closely monitor SARS-CoV-2 viral concentrations in wastewater from rural areas in New Hampshire and Vermont.

## RESULTS

**FIG 1:**
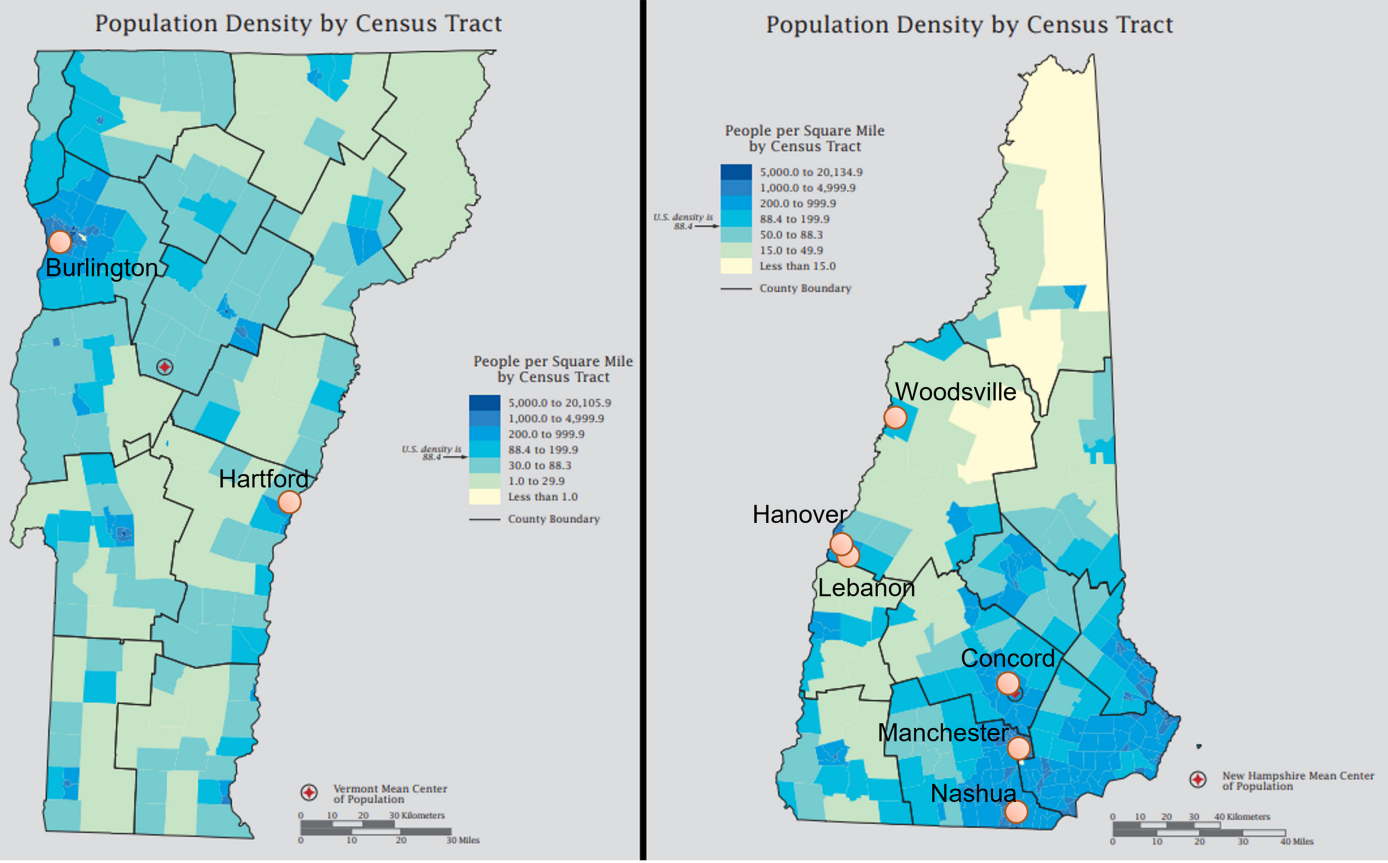
Map of municipalities in New Hampshire and Vermont. These map images were modified from the United States Census Bureau.

We received a total of 283 samples collected over a 5-month period between September 2020 and February 2021 from nine distinct WWTF in seven municipalities (Table 1, Fig. 1). Municipal sizes ranged from a population of fewer than 1,000 residents in Woodsville, NH, to nearly 90,000 residents in Nashua, NH. Sample collections began in the early fall of 2020, just prior to the regional surge in COVID-19 cases of late fall and winter 2020/2021 and ended in early February 2021 (Table S1).

**TABLE 1:** Municipality and wastewater treatment plant data and population

Site	Total no. of samples	RT-qPCR positivity, no. (%)	ddPCR positivity, no. (%)	Estimated avg flow rate per Day (MGD)	Estimated population (2019)	
Lebanon, NH	36	25 (69.4%)	28 (77.8%)	2	13,623^*a*^	
Hanover, NH	36	19 (52.8%)	21 (58.3%)	1	8,508^*a*^	
Hartford, VT	37	14 (37.8%)	19 (51.4%)	0.5	9,556^*b*^	
Woodsville, NH	39	10 (25.6%)	16 (41%)	0.025	851^*a*^	
Nashua, NH	39	36 (92.3%)	38 (97.4%)	11	88,815^*a*^	
Concord, NH	39	32 (82.1%)	35 (89.7%)	3	43,244^*a*^	
Burlington-East, VT	20	12 (60%)	11 (55%)	0.45	∼3,000	42,545^*c*^
Burlington-North, VT	21	16 (76.2%)	16 (76.2%)	1	∼10,000^*c*^	
Burlington-Main, VT	16	12 (75%)	11 (68.8%)	3.5	∼30,000	

a United States Census Bureau from 2019.

b Vermont Department of Health.

c The city of Burlington has a total population of 42,545 and the approximate number served by each WWTF are provided in the table.

**FIG 2:**
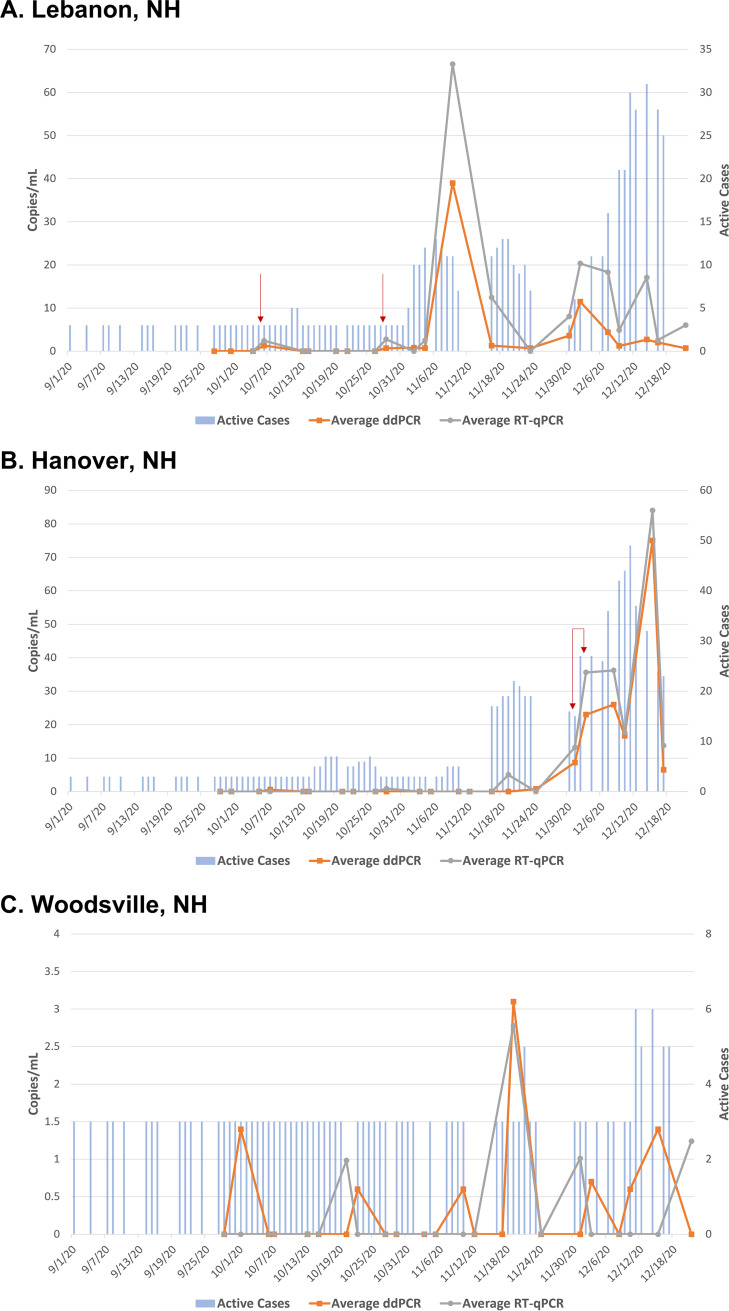
Rural municipalities. SARS-CoV-2 viral concentration in copies/mL of original wastewater sample is on the primary *y* axis, and absolute active case count data are on the secondary *y* axis. Detection methods, ddPCR and RT-qPCR, each have two targets (N1 and (N2) that are averaged and plotted as line graphs. Square point (orange) is ddPCR, circle point (gray) is RT-qPCR. Active case counts for each municipality are plotted as bar graphs over time. Any case count data below four active cases is plotted as three. Any gaps in active case count data are missing points. (A) Lebanon, NH; (B) Hanover, NH; (C) Woodsville, NH. Important dates from the results section are denoted by a red arrow.

All nine WWTF sites had at least one positive wastewater sample during the collection time, and eight out of the nine sites showed positivity in their wastewater in over 50% of the samples tested using RT-ddPCR as the detection methodology (Table 1). In general, each municipality had low active case counts during the summer months of 2020 (up through September). However, this changed significantly when active case counts hit an all-time high by midwinter. This trend was consistent throughout all monitored sites (Fig. 2 and 4, Fig. S2-S7), regardless of population density. Please note that the *y* axes vary by municipality and are not consistent between individual graphs.

### Rural municipalities.

Lebanon, NH (population 13,623, estimated wastewater flow rate of 2 MGD) had an overall SARS-CoV-2 RNA positivity rate of 77.8% using RT-ddPCR (28/36 samples) and 69.4% using RT-qPCR (25/36 samples; Table 1). Wastewater samples were largely negative through most of October, while active case counts were between three or fewer and five cases (or 0.02%–0.04% of the population) for the majority of this early fall period (Fig. 2A, Fig. S2). There was positivity in the wastewater on October 6th (denoted by a red arrow in Fig. 2A), with cases increasing to five active cases on October 11th. On October 28^th^ (also denoted by a red arrow in Fig. 2A), both RT-ddPCR and RT-qPCR showed positivity in the wastewater, which preceded the fall/winter surge. By November 2nd, case counts were at 10 cases, up from three cases or fewer on October 31^st^. Active case counts climbed to an all-time high of 31 cases by December 14th and wastewater remained positive after the positive sampling on October 28^th^.

Hanover, NH (population 8,508, estimated wastewater flow rate of 1 MGD) is home to Dartmouth College with approximately 4,500 undergraduate students. Of note, during the 2020 fall semester, only about one third of the undergraduate population were on campus in Hanover, NH. The closer approximation of Hanover, NH residents was therefore 10,000. The overall SARS-CoV-2 RNA positivity was 58.3% using RT-ddPCR (21/36 samples) and 52.8% using RT-qPCR (19/36 samples; Table 1). The trend was similar to that in Lebanon, where wastewater samples were largely negative through most of October and active case counts ranged from three or fewer to seven cases (or < 0.03%–0.06% of the population) for the majority of this early fall period (Fig. 2B, Fig. S3). Active cases rapidly increased from five on November 10th to 17 on November 16th and the positivity in the wastewater followed on November 19th (RT-qPCR, N1 = 8.36 copies/mL, N2 = 1.69 copies/mL) after being largely negative to that point. Active case counts continued to climb and reached a high of 49 cases by December 14th. This peak is linked to a facility-specific outbreak at a rehabilitation center within Hanover, NH that started the week prior. A spike in SARS-CoV-2 RNA detection in wastewater can be appreciated from December 1st (average RT-ddPCR = 8.7 copies/mL, average RT-qPCR = 13.24 copies/mL) to December 3rd (average RT-ddPCR = 23 copies/mL, average RT-qPCR = 35.62 copies/mL, denoted by bracketed arrows), coinciding with this outbreak. Wastewater remained positive after the positive sampling on November 19th for the duration of this study. While wastewater testing in Hanover, NH tracked well with active case counts, they did not prove to be a leading indicator to a rise in case counts.

Hartford, VT (population 9,556, estimated wastewater flow rate of 0.5 MGD) had an overall SARS-CoV-2 RNA positivity of 51.4% using RT-ddPCR (19/37 samples) and 37.8% using RT-qPCR (14/37 samples; Table 1). This community’s wastewater showed more sporadic positivity compared to other sites in our cohort, with negative and positive results interspersed during the regional COVID-19 surge (Fig. S4). Woodsville, NH (population 851, estimated wastewater flow of 0.25 MGD) follows a similar sporadic positivity trend (Fig. 2C, Fig. S5). Though this was the smallest municipality, the overall SARS-CoV-2 positivity was still 41% using RT-ddPCR (16/39 samples) and 25.6% using RT-qPCR (10/39 samples; Table 1). The sporadic detection of SARS-CoV-2 in the wastewater of these two small rural sites limited the utility of this type of environmental surveillance during the study period.

Of note, a significantly higher averaged N1 and N2 value was resulted on the same collection date of December 22, 2020 for Hanover and Woodsville, NH using both methodologies (Fig. S3 and S5). This date was within the winter surge and coincided with a spike in cases due to the holiday season and the facility-specific outbreak at the rehabilitation center in Hanover, NH. Though these samples were clarified and frozen on that same day of arrival, they were extracted and assayed using RT-ddPCR and RT-qPCR in different plates and on different dates.

### Urban municipalities.

**FIG 3:**
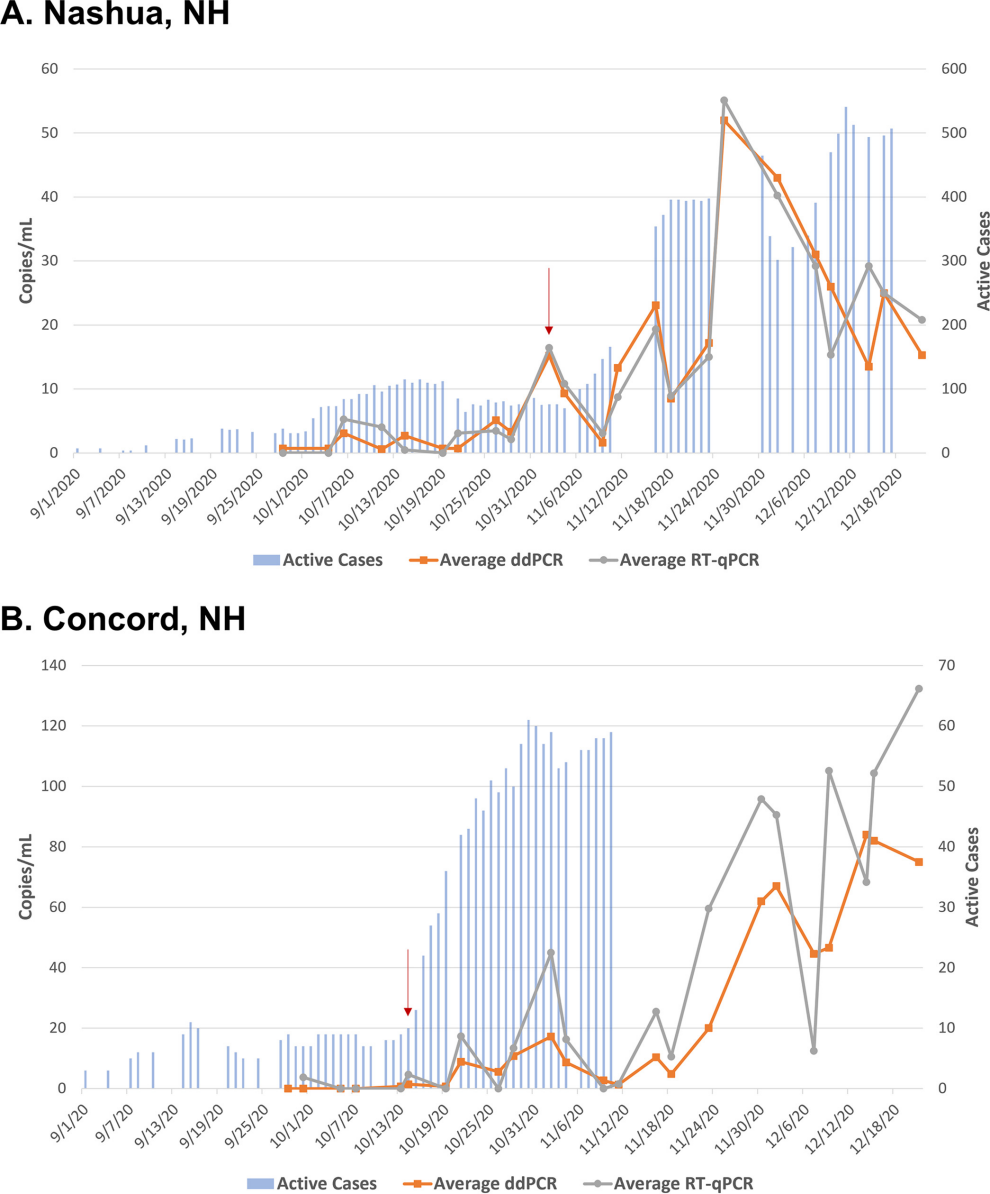
Urban municipalities. SARS-CoV-2 viral concentration in copies/mL of original wastewater sample is on the primary *y* axis, and absolute active case count data are on the secondary *y* axis. Detection methods, ddPCR and RT-qPCR, each have two targets (N1 and N2) that are averaged and plotted as line graphs. Square point (orange) is ddPCR, circle point (gray) is RT-qPCR. Active case counts for each municipality are plotted as bar graphs over time. Any case count data below four active cases is plotted as three. Any gaps in active case count data are missing points. (A) Nashua, NH; (B) Concord, NH. Important dates from the results section are denoted by a red arrow.

Nashua, NH (population 89,000, estimated wastewater flow rate of 11 MGD), the largest municipality in the study, exhibited the highest wastewater positivity rate. 97.4% of samples tested positive for SARS-CoV-2 RNA using RT-ddPCR (38/39 samples, with one sample failure) and 92.3% using RT-qPCR (36/39 samples; Table 1). Coinciding with these results, Nashua, NH had the highest active case counts from the cohort, reaching a high of 584 active cases (or 0.65% of population) on January 24, 2021, up from a low of three cases or fewer on September 8, 2020 (Fig. 3A, Fig. S6). Active case counts doubled from September 28^th^ to October 5th (38 to 73 active cases), and the wastewater showed positivity on the first sampling day of our study (September 28^th^) and again on October 5th using RT-ddPCR, though both samples were negative via RT-qPCR. Further, case counts doubled again from November 4th to November 9th (70 to 147 cases), and wastewater positivity increased in concentration by a similar factor from October 26^th^ (N1 = 7.8 copies/mL, N2 = 2.4 copies/mL) to November 2nd (N1 = 14.6 copies/mL, N2 = 15.8 copies/mL, denoted by a red arrow in Fig. 3A) using RT-ddPCR. In both doubling instances, the positivity in the wastewater occurred approximately 7 days ahead of the increase in cases.

Although Concord, NH is about half the size of Nashua, NH, at approximately 43,000 residents (estimated wastewater flow rate of 3 MGD), it is an urban center with frequent tourism and business travel. Concord, NH had a SARS-CoV-2 RNA positivity rate of 89.7% using RT-ddPCR (35/39 samples) and 82.1% of samples having positivity using RT-qPCR (32/39 samples; Table 1). Similar to other municipalities, case counts were low during the early fall with 11 or fewer active cases per day until mid-October (or < 0.03% of the population; Fig. 3B, Fig. S7). Wastewater was also largely negative during this time. By October 14th (denoted by a red arrow in Fig. 3B), SARS-CoV-2 RNA was detectable in the wastewater using both methods while case counts were still low (10 active cases). By October 18th, case counts had tripled to 29 active cases, showing that wastewater positivity was approximately 4 days ahead of this increase in cases.

### Burlington, VT.

**FIG 4:**
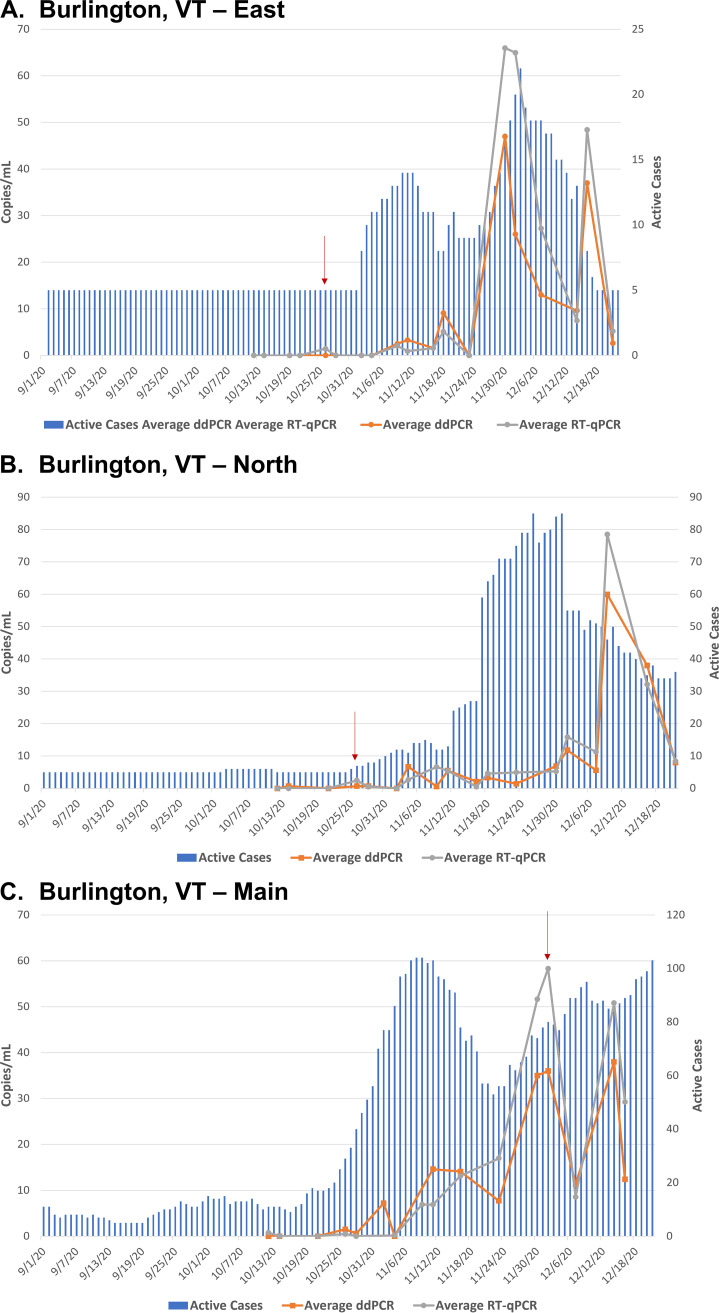
Burlington, Vermont. SARS-CoV-2 viral concentration in copies/mL of original wastewater sample is on the primary *y* axis, and absolute active case count data are on the secondary *y* axis. Detection methods, ddPCR and RT-qPCR, each have two targets (N1 and N2) that are averaged and plotted as line graphs. Square point (orange) is ddPCR, circle point (gray) is RT-qPCR. Active case counts for each municipality are plotted as bar graphs over time. Any case count data below four active cases is plotted as three. (A) East Plant, (B) North Plant, (C) Main Plant. Important dates from the results section are denoted by a red arrow.

Wastewater treatment in Burlington, VT is divided into three distinct facilities: East, North, and Main (Fig. 4). Due to this division, the estimated population that each site treats is listed in Table 1, so each facility is more comparable to a small municipality. This distinction makes Burlington, VT different than the other two larger/urban municipalities.

The East facility processes the smallest amount of wastewater per day (estimated wastewater flow rate of 0.45 MGD) and covers the smallest population of approximately 3,000 permanent residents, and closer to 5,000 residents when including a portion of the University of Vermont (UVM) students (Table 1). This facility also serves the UVM Medical Center campus. This facility had a SARS-CoV-2 RNA positivity rate of 55% using RT-ddPCR (11/20 samples) and 60% using RT-qPCR (12/20 samples). Active case counts at this site were low through October (less than six cases), while wastewater was negative (Fig. 4A). On October 26^th^ (denoted by a red arrow in Fig. 4A), the wastewater showed positivity using RT-qPCR (N1 = 0 copies/mL, N2 = 2.889 copies/mL) which preceded a doubling of cases to 10 active cases by November 2nd. Wastewater also reached a peak of SARS-CoV-2 RNA level (average RT-ddPCR = 47 copies/mL and average RT-qPCR = 65.953 copies/mL) on November 30^th^, coinciding with a peak in cases for this site of 22 cases on December 2nd.

The North facility has an estimated wastewater flow rate of 1 MGD and serves approximately 10,000 residents, including the residential New North End (Table 1). This facility had a SARS-CoV-2 RNA positivity rate of 76.2% using both RT-ddPCR and RT-qPCR (16/21 samples). The trend for the North plant is similar to the above East plant, where the active case counts were low through most of October, the doubling between October 24^th^ and October 31^st^ from fewer than six cases to 10 cases, and then up to 15 cases by November 7th (Fig. 4B). Positivity in the wastewater was first seen in the October 26^th^ (denoted by a red arrow in Fig. 4B) sample using both detection methods (average RT-ddPCR = 0.7 copies/mL and average RT-qPCR = 2.483 copies/mL), preceding the increase in cases.

The Main facility has an estimated wastewater flow rate of 3.5 MGD and covers the largest population of approximately 30,000 residents and includes the downtown business area (Table 1). This facility had a SARS-CoV-2 RNA positivity rate of 68.8% using RT-ddPCR (11/16 samples) and 75% using RT-qPCR (12/16 samples). The highest wastewater positivity level was on December 2nd (average RT-ddPCR = 36 copies/mL and average RT-qPCR = 58.320 copies/mL, denoted by a red arrow in Fig. 4C), with active cases in the 70s and 80s the week before and after this sampling. This Main site had more absolute case counts compared to the other sites, and while wastewater testing at the Main site tracked well with active case counts, they did not predict an upcoming increase in case counts.

### N1 and N2 comparison.

**FIG 5:**
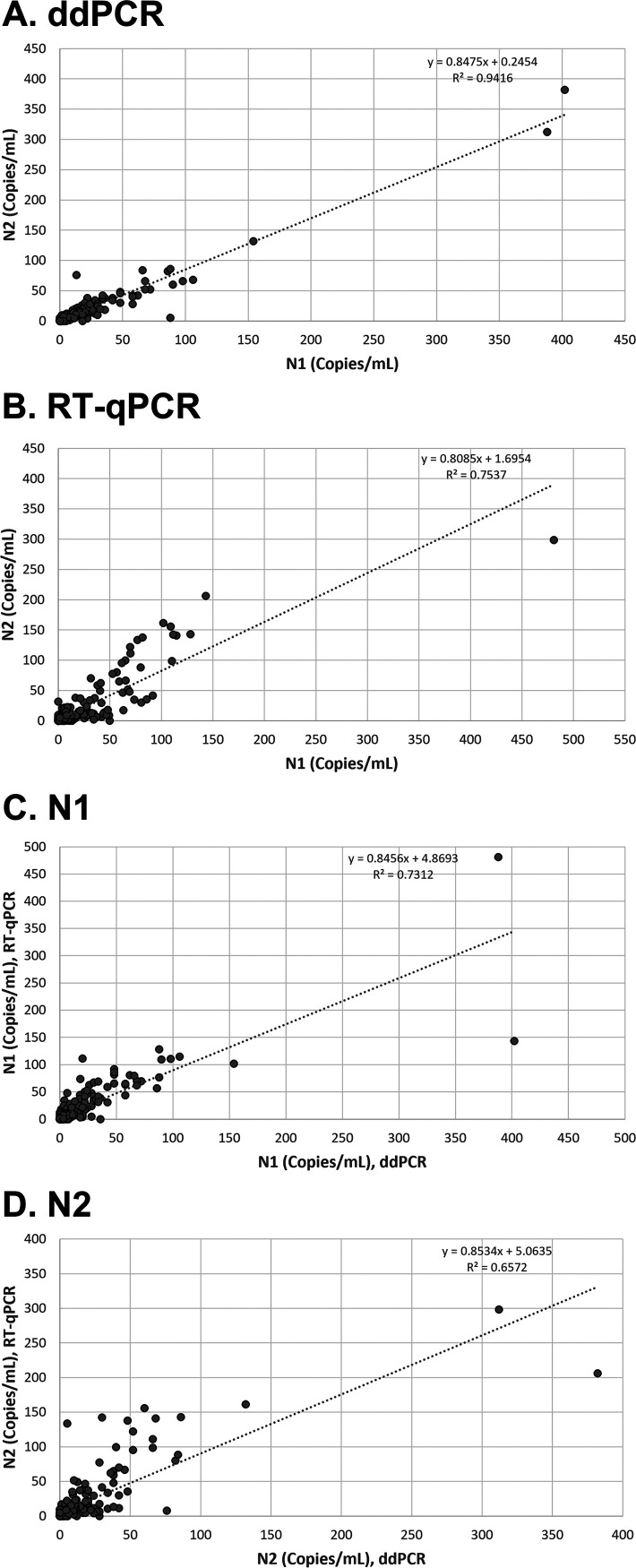
Comparison of SARS-CoV-2 targets (N1 and N2) in wastewater. (A) Comparison of N1 versus N2 for all detectable ddPCR data. (B) Comparison of N1 versus N2 for all detectable RT-qPCR data. (C) Comparison of N1 target only for ddPCR versus RT-qPCR. (D) Comparison of N2 target only for ddPCR versus RT-qPCR.

The SARS-CoV-2 N1 and N2 targets measured by RT-ddPCR correlated well to each other (R^2^ = 0.9416) within the complete data set of all municipalities (Fig. 5A). Using RT-qPCR, N1 and N2 results exhibited a lower correlation of R^2^ = 0.7537 (Fig. 5B). Fig. 5C and D compare the same target for each of the different methods. N1 demonstrated a moderate correlation between RT-ddPCR and RT-qPCR (R^2^ = 0.7312, Fig. 5C), while N2 had a slightly lower correlation (R^2^ = 0.6572, Fig. 5D).

## DISCUSSION

This study successfully collected bi-weekly wastewater samples from small cities and rural municipalities in New Hampshire and Vermont during the fall and winter of 2020/2021. The primary focus was to monitor SARS-CoV-2 viral RNA in wastewater from a region that had low active case counts during the summer and early fall of 2020 and assess whether wastewater would serve as a good alert system for an impending surge. This study was also designed to be long-term and is the longest known wastewater surveillance study performed for SARS-CoV-2 to date, testing the same WWTFs for five consecutive months. This allowed for the close monitoring of SARS-CoV-2 levels in the wastewater before and during the fall 2020/winter 2021 surge of the COVID-19 pandemic. We also contributed to the growing literature by comparing two different molecular detection methods (RT-ddPCR and RT-qPCR) and two of the CDC-designated targets to each other in over 280 real wastewater samples. We, like others, determined that monitoring SARS-CoV-2 in wastewater could serve as an early indicator preceding a surge in cases; however, this was not true at all locations monitored.

Several municipalities in our study, like Lebanon, NH, Nashua, NH, Concord, NH, and Burlington, VT (East and North), showed similar trends in wastewater positivity slightly preceding a rise in active case counts. These data are like the findings of Peccia et al. in the New Haven, CT-metro area (16), as well as Randazzo et al. in the Murcia region of Spain (18). In Lebanon, NH, the wastewater positivity preceded the increase in case counts by 5 days, while Nashua, NH and Concord, NH wastewater positivity preceded case count increases by seven and 4 days, respectively. The Burlington, VT East and North plants both showed wastewater positivity before the case count increase by as many as 7 days.

Positivity in the wastewater from Hanover, NH, was detected after a localized COVID-19 outbreak at a rehabilitation center with approximately 90% of residents testing positive by mid-December 2020 (19). Multiple employees from this rehabilitation center also tested positive, and a total of six residents died from COVID-19 due to this outbreak (20). The SARS-CoV-2 RNA level in the Hanover municipality wastewater was positive on December 1 but tripled in concentration just 2 days later on December 3. This was most likely linked to this particular facility-specific outbreak.

There were certain locations where SARS-CoV-2 RNA surveillance in wastewater did not work as well as other sites. These locations were typically the smaller municipalities with the least amount of wastewater being processed per day (in MGD, see Table 1), like Woodsville, NH and Hartford, VT. Some reasons why this might have been the case in these locations was that the population was too small and the active case counts were too low. It is likely that the SARS-CoV-2 concentration in the wastewater of these sites was hovering around the limit of detection for this assay and may explain why the results vacillate between positive and negative. It is also possible that WWTFs serving such small populations are more prone to bolus flow. For sites that are this small, sampling more frequently than twice weekly may be required to successfully monitor SARS-CoV-2 in wastewater. Alternatively, a more sensitive method may be necessary.

Though detecting SARS-CoV-2 viral RNA in a dilute sample can be difficult, we showed that concentrating clarified wastewater samples and using RT-ddPCR and/or RT-qPCR for detection resulted in a sensitive method {Robbins et al. Submitted} capable of detecting relatively small increases in active COVID-19 case counts. We used two detection methods for each sample collected during this study for results comparison. RT-ddPCR showed positivity in more samples than RT-qPCR, though it cannot be determined if RT-ddPCR showed false positivity or if RT-qPCR showed false negativity. The methods correlated moderately well with each other, with slightly higher correlation between methods for the N1 target (R^2^ = 0.7312) compared to the N2 target (R^2^ = 0.6572). N1 and N2 performed similarly well between the two assays, and while RT-ddPCR may be more sensitive, it does involve additional equipment and expertise specific to generating droplets and reading the droplet PCRs. In comparison, RT-qPCR is easier to perform and does not require any special equipment outside of a qPCR instrument. The preferred method used will depend on the resources of an individual laboratory.

To have the most community impact, it is important to promptly test and report out the wastewater sample results immediately, ideally within a 24–48 h turnaround time. This may give the community a lead of several days to enforce community safety precautions, such as quarantine and masking mandates. From our assessment, once a municipality showed positivity in the wastewater, then it generally continued to stay positive throughout the study. The highest utility for this type of surveillance testing may be regular monitoring during a period of low active case counts to alert for a potential upcoming increase in disease transmission in the community.

Though this study has many strengths, there are several key limitations. Table 1 outlines the population for each of the municipalities within this study, but the number of inhabitants served in the sewer shed of each wastewater treatment facility may not align with this stated population. Because many of the towns in our study are small and rural, many homes are not serviced by town sewer lines and instead have private septic tanks. For the purposes of this study, it was not possible to separate those on town sewer and those with their own septic tanks. Additionally, some of the treatment plants cover parts of other neighboring towns and may serve more people than just the population of the individual town. For example, the Lebanon, NH wastewater treatment facility also serves part of Enfield, NH (a neighboring town of about 4,500 people). It is also difficult to extrapolate our study further than this region due to differences in wastewater treatment plant protocols. There are over 16,000 distinct wastewater treatments in the United States alone (21), and each one has distinct procedures in place with variable handling approaches (16). In addition, there are several sewer shed variables that could affect the concentration of SARS-CoV-2 RNA in a given sample, such as total suspended solids (TSS), flow rate (in MGD), and pH. We were unable to normalize the variability to any of these factors. In addition to these variables, daily weather changes were not accounted for (22). The states of NH and VT can have drastic weather changes, including snow, rain, and dry periods. For example, there was a snowstorm in the region on December 17, 2020 which had accumulations of over three feet in some areas (23), and may have been the reason several sites (Hanover, NH and Woodsville, NH) resulted very high outlier SARS-CoV-2 RNA concentrations on December 22^nd^. The ability to use wastewater testing as a lead indicator of community spread requires rapid processing and analysis of wastewater samples in real time. Samples in this current study were frozen and analyzed in a retrospective manner. As mentioned above, a more impactful turnaround time from sample collection to results would be 24–48 h, or less if possible. Without the use of the Hamilton Microlab STAR robotics, the labor-intensive nature of the protocol used in this study would present a significant logistical limitation to achieving this turnaround time for many laboratories. The utility of wastewater testing in areas where clinical sample testing using a highly sensitive detection method (like PCR) has a fast turnaround time (<24 h) is also less compared to areas where clinical testing is lagging. Lastly, there is no guidance on the definition of a positive wastewater result and without a standardized cutoff value, it is difficult to know if the detection of virus in wastewater will predict a rise in case counts. The magnitude of change in concentration needed to determine a “rise” becomes particularly important in areas with detectable baseline concentrations of virus.

In conclusion, we showed that SARS-CoV-2 RNA positivity in wastewater precedes spikes in active cases detected in rural communities by as much as 7 days. With timely collection, analysis, and result reporting, a small community could potentially identify an impending case surge and tighten mask mandates to help mitigate community transmission of SARS-CoV-2.

## MATERIALS AND METHODS

### Sample collection.

Wastewater was collected twice per week at municipal WWTF in Lebanon, Hanover, Woodsville, Nashua, and Concord, NH as well as Hartford and Burlington (three sites: East, North, and Main), VT (Fig. 1, Table S1). Influent wastewater (prior to any treatment) was collected over a 24-h period by WWTF personnel using a composite sampler with samples chilled during the collection (24). Wastewater samples (100 mL) were transported on ice via courier to the testing laboratory for processing and analysis. Samples were collected twice weekly from September 28, 2020 through February 11, 2021 for most sites (through December 21, 2020 for Burlington, VT). WWTF personnel also collected routine data upon each sampling and provided crude estimates on average wastewater flow rate in millions of gallons per day (MGD) for each facility.

### SARS-CoV-2 active case counts.

Active SARS-CoV-2 case counts (i.e., total individuals testing positive for SARS-CoV-2 in a given community in the preceding 14 days) for the NH municipalities were collected from the NH Division of Public Health Services on the COVID-19 dashboard (https://www.covid19.nh.gov/). Exact number of active cases when fewer than 4 individuals were infected was suppressed and reported as <4 on the dashboard, and graphed as 3 in all figures in the manuscript. Hartford, VT case counts were obtained via the VT Department of Health (https://www.healthvermont.gov/covid-19). Data for active cases in Burlington, VT were obtained stratified by sewer shed from the VT Department of Health.

### Wastewater sample processing.

A summary of the methods can be seen in Fig. S1. Briefly, 45 mL of wastewater samples were centrifuged at 4,600 × *g* at 4°C for 30 min and 40 mL of supernatant (“clarified” sample) was transferred to new tubes. Samples were frozen at −80°C for up to 3 months before use. Upon thawing, 10% polyethylene glycol 8000 (PEG) and 2.25% wt/vol NaCl were added (25, 26), and samples were centrifuged at 12,000 × *g* at 4°C for 2 h. The pellets were re-suspended in 800 μL nuclease-free water and 200 μL of this suspension was then used for RNA extraction using the Promega Wastewater Large-Volume TNA Capture kit (50 μL elution volume, Catalog No. AX9550, Madison, WI, USA) with a magnetic bead-based protocol automated on a robotic liquid handling system with 96-well plate capacity (Microlab STAR, Hamilton Company, Reno, NV, USA).

### Detection methods.

For reverse transcriptase-quantitative PCR (RT-qPCR) method, the Promega Wastewater SARS-CoV-2 RT-qPCR Kits (Catalog No. CS317409and CS317410, Promega, Madison, WI, USA) were used to detect the N1 and N2 nucleocapsid gene targets (FAM-labeled probes) developed by the CDC along with a process control (Pepper Mild Mottle Virus, PMMV, CY5-labeled probe) and an internal amplification control (HEX-labeled probe). In addition, a no template control (NTC), genomic RNA from SARS-CoV-2 (Isolate USA-WA1/2020, NR-52285, BEI Resources, Manassas, VA, USA) diluted in nuclease-free water (1:100 concentration, 5.5 × 10^6 GE/mL [2,750GE/reaction]), and an extraction NTC were used. A serial dilution of the quantification standard dsDNA included in the Promega RT-qPCR kit was run in duplicate replicate on each plate to create a calibration curve for each gene target. Standard curves were acceptable if they had a slope between −3.1 to −3.6, an efficiency value >90%, and an R^2^ value >0.99. Valid internal controls required Cq values between 20 and 25 and PMMV Cq values between 20 and 30 cycles. The PCR conditions were as follows: 15 min at 45^0^C, 2 min at 95^0^C, 40 cycles of 95°C denaturation for 3 s and 62°C annealing for 30 s.

For reverse transcriptase-droplet digital PCR (RT-ddPCR) method, we used the Taq-Man PCR mixture and probe from Bio-Rad Laboratories (Hercules, CA, USA). Controls used included: NTC, genomic RNA from SARS-CoV-2 diluted in nuclease-free water (Isolate USA-WA1/2020, NR-52285, BEI Resources, Manassas, VA, USA; 1:100 concentration, 5.5 × 10^6 GE/mL (or 2,750 GE/Reaction)), genomic RNA from SARS-CoV-2 diluted in negative wastewater (post-extraction, 1:100 concentration), and an extracted NTC. Droplets were generated on the Automated Droplet Generator (Bio-Rad Laboratories, Hercules, CA, USA) and droplet-portioned samples were cycled in a thermal cycler (Bio-Rad Laboratories, Hercules, CA, USA) with the following protocol: 60 min at 50°C (reverse transcription step), 10 min at 95^0^C, 40 cycles of 94°C denaturation for 30 s and 55°C annealing for 30 s, followed by 10 min at 98^0^C, and then hold at 4^0^C. Plates were then read on the Bio-Rad QX200 Droplet Reader (FAM and HEX).

### Summary of wastewater SARS-CoV-2 detection validation.

The wastewater detection protocol for both RT-qPCR and RT-ddPCR was validated by establishing linearity, precision, and LOD {Robbins et al. Submitted}. Linearity of both RT-qPCR and RT-ddPCR assays was demonstrated using a 7-fold serial dilution of the BEI control RNA spanning concentrations of 5.5 × 10^7^ to 5.5 × 10^1^ genomic equivalents (ge)/mL diluted in both nuclease free water (R^2^ = 0.99) and previously established extracted negative wastewater (R^2^ = 0.97). The AccuPlex SARS-CoV-2 Verification Panel (LGC SeraCare, Milford, MA), including three concentrations of whole-genome SARS-CoV-2 viral-based reference material (3, 4, and 5 log copies/mL) were either directly extracted or spiked into clarified wastewater, PEG concentrated, and then extracted. Each concentration was detected on both RT-qPCR and RT-ddPCR (*n* = 5), and the linearity equations for both PCR assays and both N1 and N2 targets had slopes between 1.0 and 1.2 with R^2^ values greater than 0.96. To determine the limit of detection (LOD), SeraCare Calibration material was diluted in SARS-CoV-2 negative clarified wastewater at concentrations of 50, 100, 250 and 500 copies/mL and extracted in replicate (*n* = 24). The LOD for the study with detection in greater than 95% of replicates was 100 copies/mL for both detection methods. Analytical specificity could not be determined due to the lack of a reference method and standardized testing guidelines for wastewater testing and surveillance.

### Data analysis.

Quantification of N1 and N2 RNA (copies/mL) was determined for each sample by both RT-qPCR and RT-ddPCR using the calibration curve created with each run of the RT-qPCR assay and absolute quantification by RT-ddPCR, respectively. For RT-ddPCR, one droplet containing either the SARS-CoV-2 N1 or N2 target was required to be considered positive. Original sample concentration (N1 and N2 copies/mL of original wastewater sample) was back-calculated assuming 100% recovery at each step of the concentration and RNA extraction protocol. N1 and N2 concentrations were averaged together for each sample for the purposes of data visualization and graphing. Wastewater concentrations were plotted against active case counts for each municipality. Active case counts that were below four cases were graphed as three (including zero cases).
