# Supplementary material for: Wastewater-Based SARS-CoV-2 Surveillance in Northern New England
Source: Microbiol Spectr. 2022 Apr 12;10(2):e02207-21. doi: 10.1128/spectrum.02207-21 (PMC9045146; doi:10.1128/spectrum.02207-21)
Supplement: SUPPLEMENTAL FILE 1 — Supplemental material. Download spectrum02207-21-s001.pdf, PDF file, 0.7 MB [file spectrum02207-21-s001.pdf]

Figure S1:

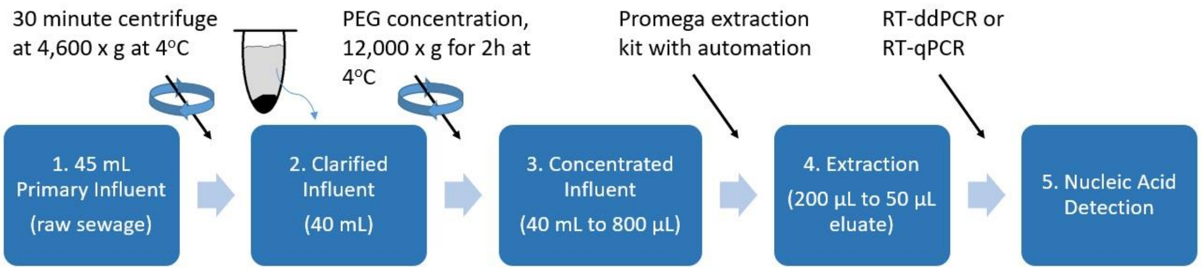

Figure S1: Summary of Methods. 1. 45 mL of primary influent is centrifuged at 4,600 x g at 4°C for 30 minutes in a 50 mL conical tube. 2. About 40 mL of clarified supernatant is then moved to a new 50 mL conical tube, which is then PEG concentrated (with 10% PEG and 2.25% w/v NaCl, centrifuged at 12,000 x g at 4°C for 2 hours). 3. The supernatant is then removed, and the pellet is resuspended in 800 µl of nuclease free water. 4. 200 µl of this concentrated sample is then processed using the Promega extraction kit with Hamilton STAR automation, which results in 50 µl of eluate. 5. An aliquot of this eluate is then used for each nucleic acid detection method (RT-ddPCR and RT-qPCR).

Figure S2:

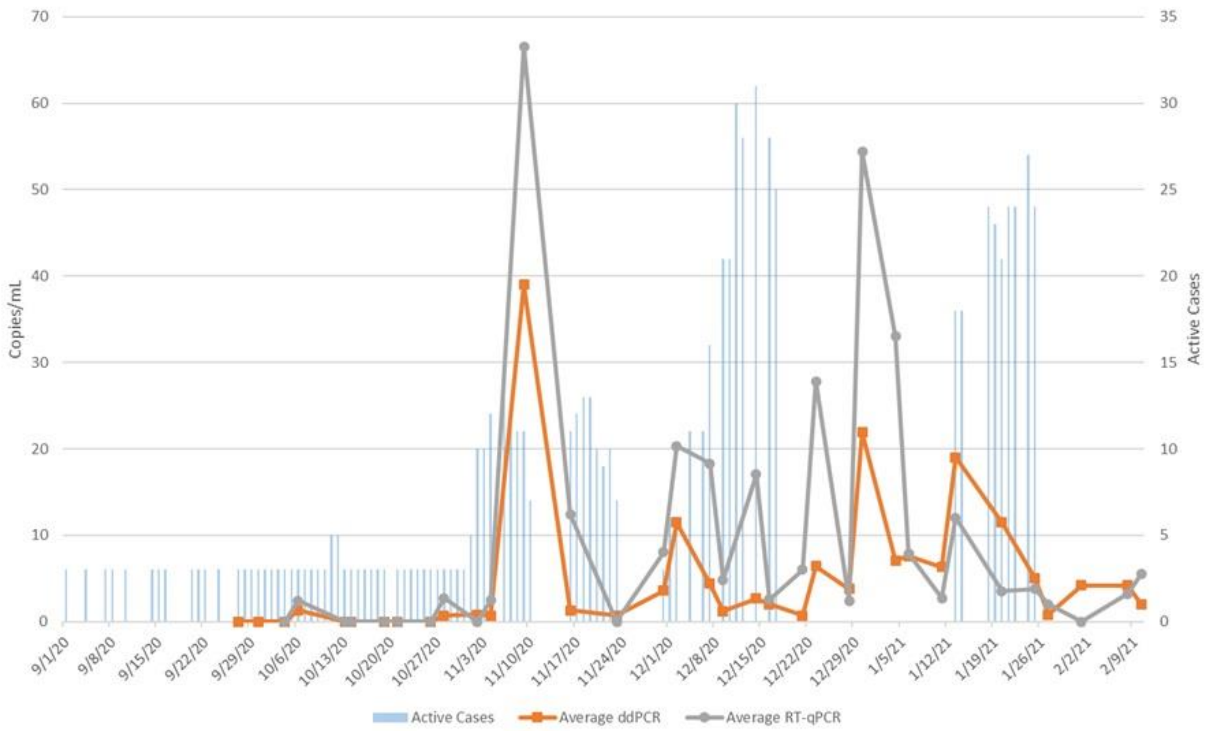

Figure S2: All Data Points for Lebanon, NH. SARS-CoV-2 viral concentration (copies/mL) of original wastewater sample (left y-axis) and absolute active case count data (right y-axis) plotted over time in Lebanon, NH. Detection methods, ddPCR (orange squares) and RT-qPCR (gray circles), each have two targets (N1 and N2) that were averaged and plotted as line graphs. Active case counts for each municipality are plotted as bar graphs over time. Any case count data below four active cases is plotted as three. Any gaps in active case count data are missing points.

Figure S3:

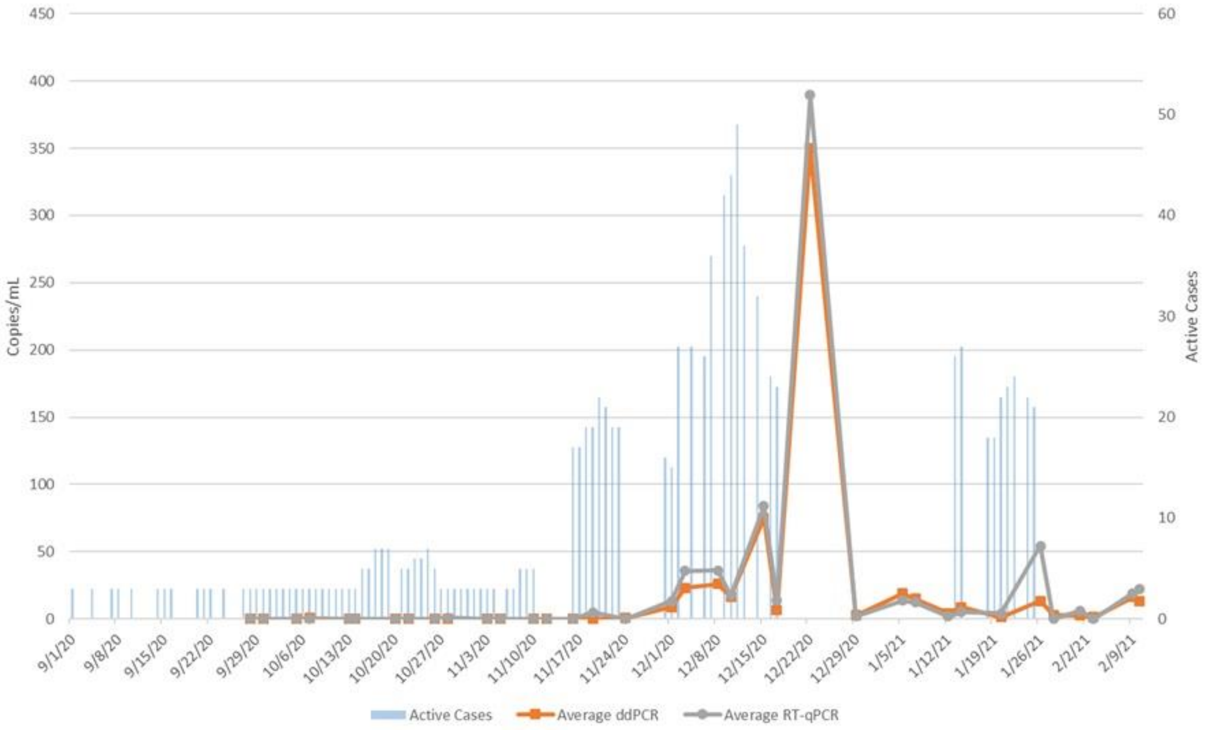

Figure S3: All Data Points for Hanover, NH. SARS-CoV-2 viral concentration (copies/mL) of original wastewater sample (left y-axis) and absolute active case count data (right y-axis) plotted over time in Hanover, NH. Detection methods, ddPCR (orange squares) and RT-qPCR (gray circles), each have two targets (N1 and N2) that were averaged and plotted as line graphs. Active case counts for each municipality are plotted as bar graphs over time. Any case count data below four active cases is plotted as three. Any gaps in active case count data are missing points.

Figure S4:

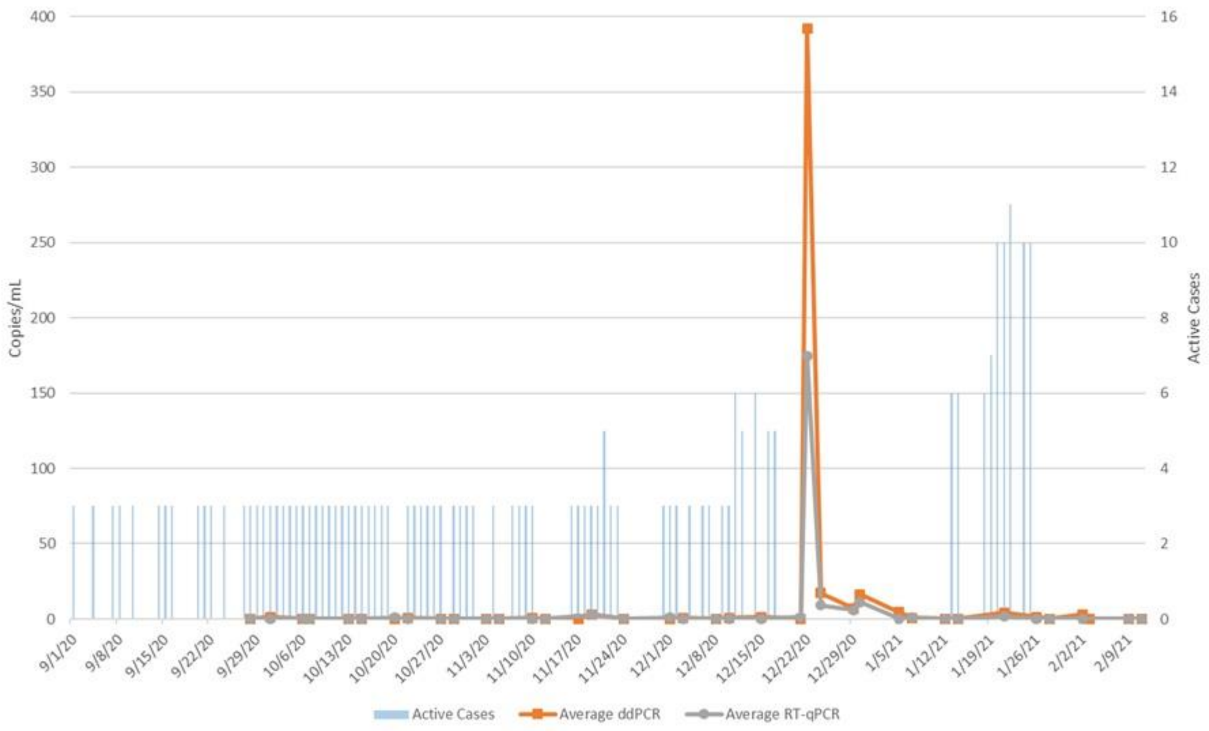

Figure S4: All Data Points for Woodsville, NH. SARS-CoV-2 viral concentration (copies/mL) of original wastewater sample (left y-axis) and absolute active case count data (right y-axis) plotted over time in Woodsville, NH. Detection methods, ddPCR (orange squares) and RT-qPCR (gray circles), each have two targets (N1 and N2) that were averaged and plotted as line graphs. Active case counts for each municipality are plotted as bar graphs over time. Any case count data below four active cases is plotted as three. Any gaps in active case count data are missing points.

Figure S5:

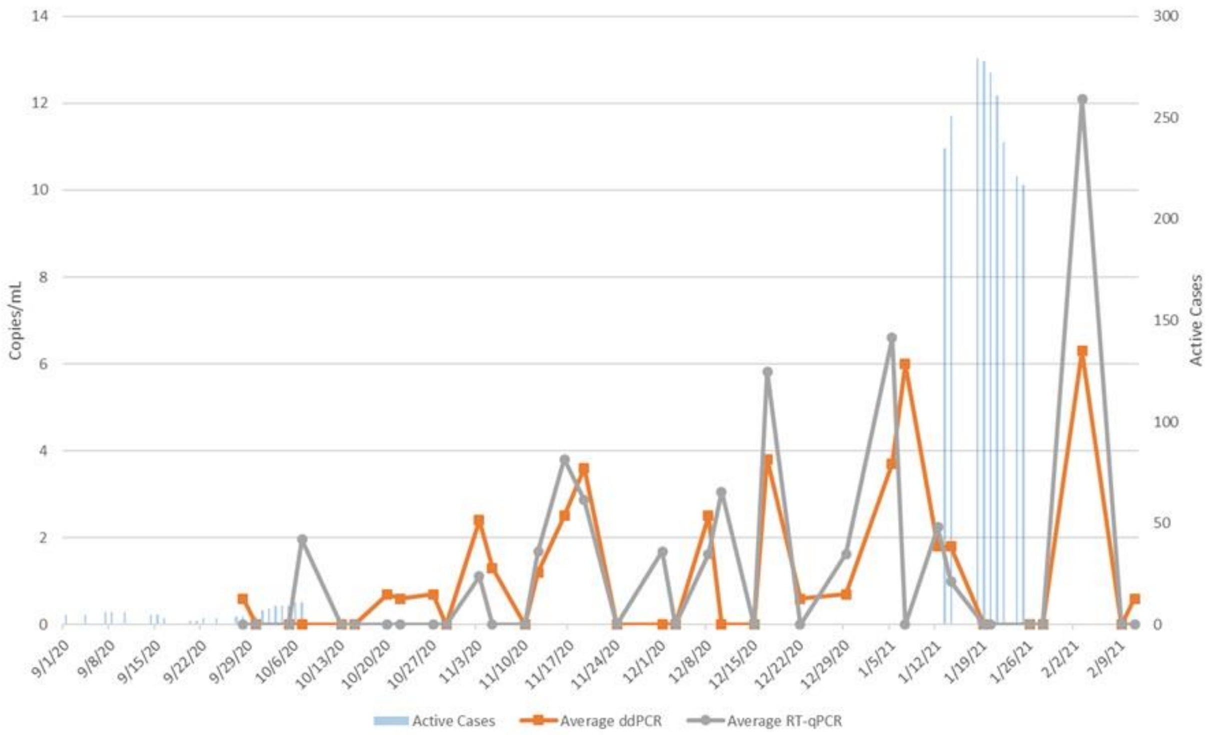

Figure S5: All Data Points for Hartford, VT. SARS-CoV-2 viral concentration (copies/mL) of original wastewater sample (left y-axis) and absolute active case count data (right y-axis) plotted over time in Hartford, VT. Detection methods, ddPCR (orange squares) and RT-qPCR (gray circles), each have two targets (N1 and N2) that were averaged and plotted as line graphs. Active case counts for each municipality are plotted as bar graphs over time. Any case count data below four active cases is plotted as three. Any gaps in active case count data are missing points.

Figure S6:

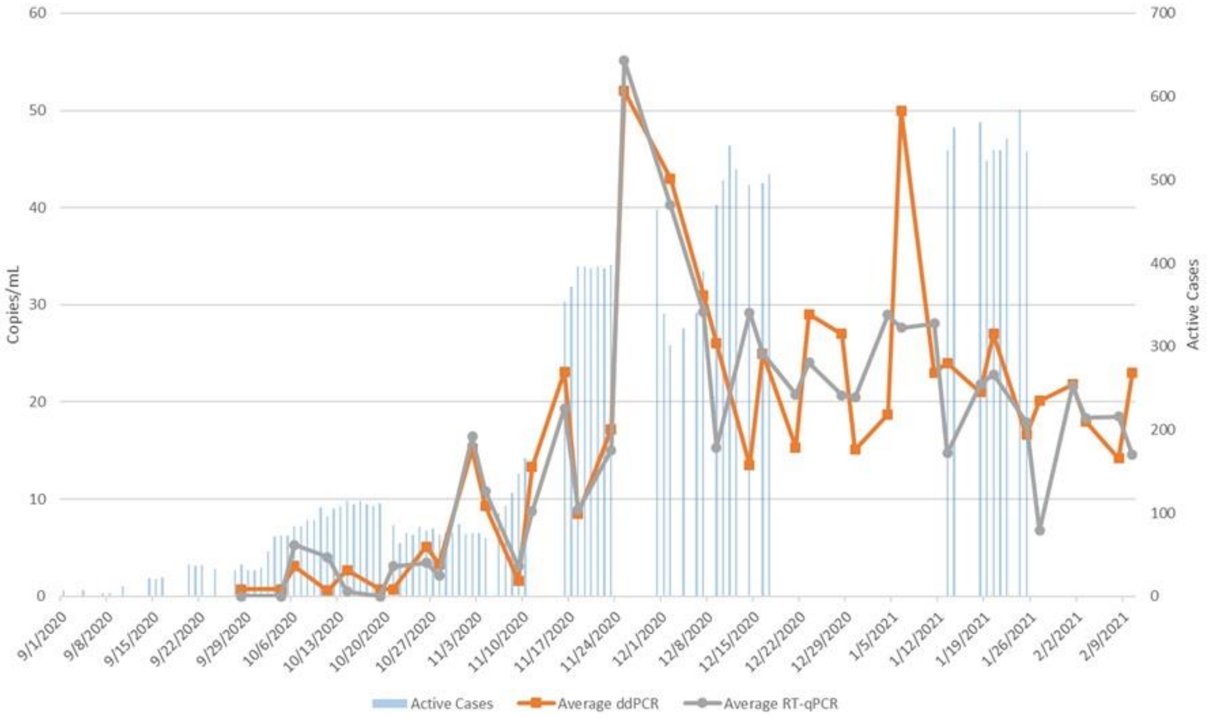

Figure S6: All Data Points for Nashua, NH. SARS-CoV-2 viral concentration (copies/mL) of original wastewater sample (left y-axis) and absolute active case count data (right y-axis) plotted over time in Nashua, NH. Detection methods, ddPCR (orange squares) and RT-qPCR (gray circles), each have two targets (N1 and N2) that were averaged and plotted as line graphs. Active case counts for each municipality are plotted as bar graphs over time. Any case count data below four active cases is plotted as three. Any gaps in active case count data are missing points.

Figure S7:

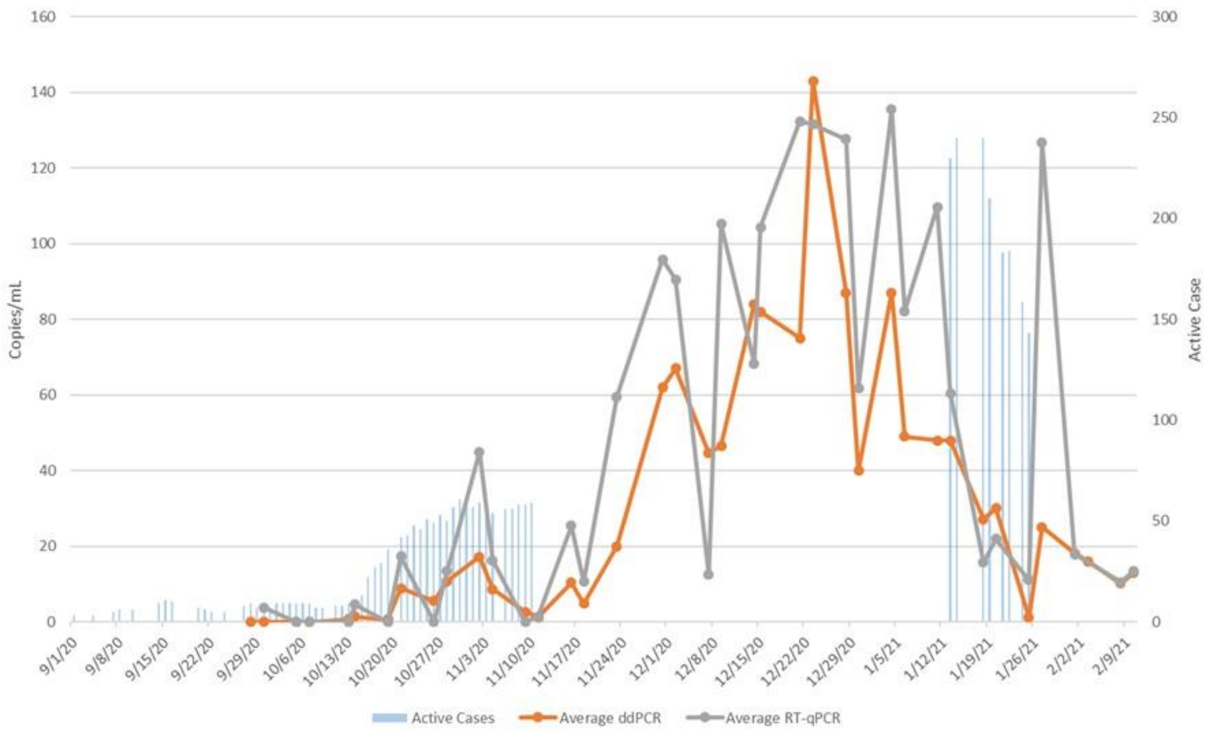

Figure S7: All Data Points for Concord, NH. SARS-CoV-2 viral concentration (copies/mL) of original wastewater sample (left y-axis) and absolute active case count data (right y-axis) plotted over time in Concord, NH. Detection methods, ddPCR (orange squares) and RT-qPCR (gray circles), each have two targets (N1 and N2) that were averaged and plotted as line graphs. Active case counts for each municipality are plotted as bar graphs over time. Any case count data below four active cases is plotted as three. Any gaps in active case count data are missing points.

Supplemental Table S1:

Site	Collection Day of the Week	First Collection Day	Last Collection Day
Lebanon, NH	M, W	9/28/2020	2/10/2021
Hanover, NH	Tu, Th	9/29/2020	2/11/2021
Hartford, VT	Tu, Th	9/29/2020	2/11/2021
Woodsville, NH	Tu, Th	9/29/2020	2/11/2021
Nashua, NH	M, W	9/28/2020	2/10/2021
Concord, NH	M, W	9/28/2020	2/10/2021
Burlington-East, VT	M, W	10/12/2020	12/21/2020
Burlington-North, VT	M, W	10/12/2020	12/21/2020
Burlington-Main, VT	M, W	10/12/2020	12/16/2020

Supplemental Table S1: Collection Dates for Each Wastewater Treatment Facility.
